# Wine psychology: basic & applied

**DOI:** 10.1186/s41235-020-00225-6

**Published:** 2020-05-13

**Authors:** Charles Spence

**Affiliations:** Department of Experimental Psychology, Crossmodal Research Laboratory, Anna Watts Building, University of Oxford, Oxford, OX2 6GG UK

**Keywords:** Wine, Experiential marketing, Packaging, Glassware, Crossmodal correspondences, Sonic seasoning

## Abstract

Basic cognitive research can help to explain our response to wine, and the myriad factors that affect it. Wine is a complex, culture-laden, multisensory stimulus, and our perception/experience of its properties is influenced by everything from the packaging in which it is presented through the glassware in which it is served and evaluated. A growing body of experiential wine research now demonstrates that a number of contextual factors, including everything from the colour of the ambient lighting through to background music can exert a profound, and in some cases predictable, influence over the tasting experience. Sonic seasoning - that is, the matching of music or soundscapes with specific wines in order to accentuate or draw attention to certain qualities/attributes in the wine, such as sweetness, length, or body, also represents a rapidly growing area of empirical study. While such multisensory, experiential wine research undoubtedly has a number of practical applications, it also provides insights concerning multisensory perception that are relevant to basic scientists. Furthermore, the findings of the wine research are also often relevant to those marketers interested in understanding how the consumers’ perception of any other food or beverage product can potentially be modified.

## Significance statement

This review paper highlights how basic cognitive research can help understand, and thereafter modify the way in which people respond to wine. There is far more research on the influence of cognitive/perceptual factors on the purchasing and the subsequent tasting experience in the world of wine than for any other food or beverage product. Hence, understanding what research has already been conducted to influence the perception of the wine itself, and the wine-drinking experience more generally, typically provides an excellent starting point as far as considering how our perception/experience of any other food or beverage product might potentially be modified as well. The review highlights a number of examples of how use-inspired basic research has already provided insights and opportunities that have been taken up by those making and/or marketing wine (e.g., in terms of the design of cellar-door experiences, multisensory experiential wine-tasting events, not to mention wine labels). For instance, over recent decades there has been a great deal of research into the importance of and expectations set by the colour of wine. Bottles, labels, brands, and glassware for wine have also been widely studied by both marketers and sensory scientists. The last decade or two has also seen something of an explosion of interest in the way that people match music with wine, and how listening to the former can transform the wine taster’s experience of the latter. Wine is an especially intriguing product to work with given its complexity and constant evolution. Ultimately, though, this can also make working with wine more challenging than with other food and beverage products.

## Introduction

Traditionally and understandably, research in the world of wine has tended to focus on oenology, viticulture, and wine sensory analysis (e.g., Amerine & Roessler, 1976; Goode, 2005, 2016; Peynaud, 1984, 1987; Zoecklein, Fugelsang, Gump, & Nury, 1995). As such, there has perhaps been little of relevance to the readership of a journal such as *Cognitive Research: Principles and Implications* (*CRPI*). There has, though, in recent decades been something of an explosion of interest in what might be termed “wine psychology”. The emerging body of research has led to a growing awareness of just how much a wide variety of cognitive and perceptual factors, what Rozin (2006) calls processes or entities (such as learning, sensation, attention, and memory) influence the wine-drinking experience, both in wine experts and regular consumers alike. The perception of wine itself and the wine-tasting experience more generally have been shown to be influenced by everything from the weight of the wine bottle through to the sound made by its closure and the glass from which it is drunk, to the wine’s visual appearance and the multisensory environment/atmosphere in which it happens to be consumed. Researchers working in a diverse range of disciplines from marketing to sensory science, and from cognitive neuroscience to packaging design and economics have started to become increasingly interested in the consumer’s response to wine, be it in terms of the latter’s purchase behaviour or their perceptual response on tasting a wine.

There is undoubtedly a great deal of psychology in the world of wine appreciation not to mention wine choice (e.g., Batt & Dean, 2000; Beverland, 2006; Bruwer, Chrysochou, & Lesschaeve, 2017; Edwards & Spawton, 1998; Escobar, Kallas, & Gil, 2018; Horska, Bercik, Krasnodebski, Matysik-Pejas, & Bakayova, 2016; Hughson, 2008; Mitchell & Mitchell, 2009). Wine writers (Goode, 2007; Smith, 2017) and cognitive neuroscientists (Shepherd, 2015, 2017) have been interested in trying to uncover the various brain areas/networks involved in wine appreciation. Indeed, according to eminent North American neuroenologist Gordon Shepherd, drinking wine “engages more of our brain than any other human behaviour” (as quoted in Hoyle, 2017). To date, the majority of the neuroimaging research has tended to focus on how the brain response changes as a result of increasing wine expertise (e.g., Banks et al., 2016; Castriota-Scanderbeg et al., 2005; Pazart, Comte, Magnin, Millot, & Moulin, 2014). Meanwhile, a separate fruitful line of neuroimaging research has looked at the effects of pricing/branding information on the brain’s response to wine in regions such as the orbitofrontal cortex (Alvino, van der Lubbe, Joosten, & Constantinides, in press; Plassmann, O’Doherty, Shiv, & Rangel, 2008; Plassmann & Weber, 2015; Schmidt, Skvortsova, Kullen, Weber, & Plassmann, 2017).

Of all the food and beverage products that could potentially be studied, it is wine that has received by far the most research interest over the last half century or so. In fact, no matter whether one is talking about the impact of colour, glassware, packaging, branding, label design, closure type, pricing, or perceptual expertise, there is just so much more research in the world of wine than in any of the world’s other more popular drinks such as, for example, coffee, tea, beer, or water (e.g., Charters & Pettigrew, 2003, 2005; D’Alessandro & Pecotish, 2013; Goldstein et al., 2008; Kidd, 1999; Lange, Martin, Chabanet, Combris, & Issanchou, 2002; Lecocq, Visser, Lecocq, & Visser, 2006; Lockshin, Jarvis, d’Hauteville, & Perrouty, 2006; Mitchell & Greatorex, 1989; Puyares, Ares, & Carrau, 2010; Siegrist & Cousin, 2009; Spence, 2019c; Spence & Wang, 2019; Tootelain & Ross, 2000; Verdú Jover, Llorens Montes, Fuentes, & M. del M., 2004; Vollherbst & Urben, 2011; Wansink, Payne, & North, 2007; see Spence, 2010c, 2014b, for reviews).

Researchers have also used wine to address questions related to local versus global information processing (Lewis, Seeley, & Miles, 2009) and verbal overshadowing (Melcher & Schooler, 1996). One of the questions that will be returned to later concerns the extent to which findings, principles, and cognitive mechanisms implicated in our relationship to wine also explain our responses to other food and drink products. As I argue later, while there are a large number of similarities there are also a few important differences.

Ultimately, I will also return to the question of what implications the study of wine psychology may have for the basic researcher interested in multisensory perception. The research outlined here is organized around one particular domain of lived everyday experience. At this point, it is perhaps worth drawing attention to a 2006 article in which Paul Rozin highlights the importance of domain-based psychology (i.e., research that is based on studying domains of everyday experience such as eating, driving, and working), rather than the dominant approach within the field of experimental psychology that is organized in terms of specific processes, or mental entities, such as learning, sensation, attention, or memory (Rozin, 2006). Rozin stresses both the contemporary neglect of domains of experience in applied psychology, but also its importance in terms of understanding our lived mental life - what he refers to as “domain denigration and process preference”.

The context, or atmosphere, in which a wine happens to be consumed also turns out to have a profound effect on the tasting experience, as acknowledged by discussion of *the Provencal rosé paradox* (e.g., Gregory, 2007; Smith, B. C. (2009). The emotional impact of a wine and the Provencal rose paradox. Unpublished manuscript; Spence, 2017a; Spence & Piqueras-Fiszman, 2014). The latter name given to the common experience that a wine that tastes delightful while on holiday often tastes very different, and is much less enjoyable, when sampled at home after one’s return. In recent years, many people have also become interested in the crossmodal correspondences that exist between wine and music, and how they can be leveraged to enhance the wine-drinking experience of regular consumers (see Spence, 2019b; Spence & Wang, 2015a, 2015b, 2015c, for reviews). The latter, an area of research falling under the name of “sonic seasoning” (Spence, 2017b), or, more particularly, when it deals with wine, “oenosthesia” (see Burzynska, 2018).

### On the importance of the visual appearance of wine

That colour influences aroma, taste, and flavour has long been known in the world of wine (Pokorný, Filipů, & Pudil, 1998; see Spence, 2010a, 2010b, for reviews). However, what has taken many people by surprise is just how easy it is to *fool* even the wine experts simply by deliberately miscolouring a wine (e.g., see Pangborn, Berg, & Hansen, 1963, for early work, and Morrot, Brochet, & Dubourdieu, 2001; Parr, White, & Heatherbell, 2003; Wang & Spence, 2019a, for more recent research). In what is by far the largest, if by no means the first, study of its kind, Wang and Spence presented 168 individuals with a white wine, a rosé wine, and a white wine that had been artificially coloured with an odourless, tasteless food dye to look exactly like the rosé wine. The participants (including 22 beginners, defined as “I drink socially but don't know much about wine”, 62 intermediates, defined as “I know which wines I like and have been to some classes”, 79 experts, defined as “I work in the wine trade and/or have 5+ years experience tasting wine formally”, and 5 not declared) were provided with an extensive list of possible wine-relevant flavour descriptors to choose from and had to provide three aroma and flavour descriptors to best describe each of the three wines. The participants were also instructed to rate their liking of the wine, the flavour intensity, and how difficult they found it to pick relevant descriptors for each wine. The participants were not informed of the visual deception.

Linguistic analysis of the results demonstrated that those participants with wine-tasting experience judged the fake rosé wine to be much more similar to the rosé wine than to the white wine, even though the fake rosé and the white wine were, in fact, one and the same. Moreover, red fruit descriptors were attributed to both rosé wines, especially in terms of flavour descriptors. Quantitative ratings revealed that the fake rosé wine was liked less than either of the authentic wines, and the participants found it more difficult to describe the fake than the authentic rosé wine. These results therefore demonstrate that while participants found the fake rosé somehow different from the two unadulterated wines, they nevertheless used the red fruit terms to describe its aroma and flavour. What is more, and as has been noted previously (see Parr et al., 2003), the experienced tasters appeared to be more influenced by colour than the non-expert participants. This is presumably because the subtle gradations of colour in wine have more meaning, and hence set more specific flavour expectations, for experts than for the beginners (i.e., the novice, or social wine drinkers). After all, the latter may only really meaningfully discriminate between broad categories including red, white, rosé, and, these days, possibly also orange wines (see also Manescu et al., 2018).^1^

There is a much richer range of colours in the world of wine than is the case for perhaps any other class of beverage (see Spence, 2010b). Furthermore, wine experts are trained to carefully assess the colour of a wine first when tasting (see Amerine & Roessler, 1976; Gawel, 1997; Jackson, Timberlake, Bridle, & Vallis, 1978; Spence, 2014a; see also Coulon-Leroy, Pouzalgues, Cayla, Symoneaux, & Masson, 2018). Research from Ballester, Abdi, Langlois, Peyron, and Valentin (2009) has shown that wine experts can correctly group red and white wines on the basis of their aroma when served in black tasting glasses (to obscure any visual cues). However, they struggle when it comes to categorizing rosé wines correctly (see also Valentin, Parr, Peyron, Grose, & Ballester, 2016).

The clarity/limpidity is another important aspect of a wine’s visual appearance. However, to date, this has attracted far less research interest (though see Barnett, Juravle, & Spence, 2017, for equivalent research from the world of beer). Experienced wine tasters tend to focus on the colour, intensity, and clarity of a wine when evaluating its visual appearance. The famous French oenologist, Emile Peynaud (1987), p. 31, talks of a wine’s limpidity (dullness, brilliance) and colour (intensity and shade). The intensity of a wine’s appearance is described in terms of simple adjectives such as pale, light, and weak at one end of the spectrum through to deep, dark, and intense at the other. To the knowledgeable wine taster, intensity can indicate climatic conditions - with warmer years and warmer regions producing wines with deeper intensity; age - white wines pick up a deeper colour as they age, whereas red wines tend to go paler; and grape variety - where thicker-skinned red grapes tend to produce wines with a deeper colour (see Schuster, 2002). The shade of colour in a glass of wine can itself also play a role. For instance, a bright slightly-blueish tint in a red wine might indicate its youthfulness, whereas mature reds tend to take on garnet and tawny hues (Fielden, 2009; Spence, 2010b).

Finally, here, while changing the colour of the wine may well have the largest influence on the tasting experience, as we will see later, everything from the colour of the wine label (Lick, König, Kpossa, & Buller, 2017; and see Barnett & Spence, 2016; Sugrue & Dando, 2018, for similar results from the worlds of beer and cider, respectively) through to the colour of the wine glass itself (Williams, Langren, & Noble, 1984; Williams, Langren, Timberlake, & Bakker, 1984), or even the colour of the environment in which a wine is evaluated (Spence, Velasco, & Knoeferle, 2014) has now been shown to bias people’s wine expectations/judgements.

Interim summary: applied research that has highlighted the importance of colour to wine appreciation is of relevance to the basic researcher inasmuch as it provides additional support for colour-in-context theory (e.g., Elliot & Maier, 2012). What is more, wine expertise also appears to result in a greater visual dominance, or biasing, of perception. This result, then, contrasts with other areas of multisensory perception research, such as those older studies of potters, where expertise was shown not to impact the patterns of visual dominance that were observed in a visual-tactile shape task (Power & Graham, 1976; though see also Shankar, Simons, Shiv, McClure, & Spence, 2010). One other important point that is emphasized by the research around wine colour concerns just how much prediction (call it crossmodal mental imagery or expectation) occurs prior to tasting, based in the wine expert at least, on very subtle variations of hue and clarity/limpidity (e.g., Piqueras-Fiszman & Spence, 2015; Shankar, Levitan, & Spence, 2010; Spence & Wang, 2018a).

### Complexity, perceptual learning, and the perils of blind wine-tasting

The results of a number of studies conducted over the last couple of decades or so have demonstrated that even wine experts are unable to correctly judge many of the attributes of wines that wine writing would suggest that they ought to be able to discern from the chemosensory properties of the wine itself, i.e., when tasted blind (e.g., Harrar, Smith, Deroy, & Spence, 2013; Smith, 2008; Weil, 2005, 2007; Weinberg, 2008; see Spence, 2010c, for a review). For instance, Harrar et al. conducted research with 15 tasters, comprising 4 experts, 6 intermediates, and 5 novice champagne tasters, in which seven sparkling wines were presented blind. The participants were only informed that the sparkling wines (6 champagnes and one English sparkling wine) could potentially span the full range from 0 to 100% Chardonnay white grapes but were otherwise given no information about the wines that they were tasting. Furthermore, all visual cues were also obscured by presenting the sparkling wines in opaque black tasting glasses.

The tasters were instructed to try and estimate the proportion of white grapes in each of the wines while also rating their hedonic response. The wines varied from a 100% Blanc de blancs (made with 100% white Chardonnay grapes) through to a 100% Blanc de noirs (made with 100% red Pinot noir and/or Pinot Meunier grapes). In fact, the percentage of white grapes in the sparkling wines was varied systematically, with the wines included in the study made from 0%, 22%, 30%, 45%, 58%, or 100% Chardonnay grapes. None of the participants in Harrar et al.’s (2013) study were able to correctly judge the percentage of white grapes in the wines (cf. Ballester et al., 2009). What is more, the tasters’ hedonic ratings of the wines were not correlated with the price of the sparkling wines either, despite the fact that the wines varied from around £18 to an “eye-watering” £400 a bottle for the most expensive.^2^

Elsewhere, Wang and Spence (2019b) assessed whether tasters (41 novice, 30 intermediate, and 16 expert tasters by self-report) were able to detect the chemical complexity of wine by trying to identify the blends from a selection of six wines tasted blind (see Singleton & Ough, 1962, for the original inspiration behind this particular study). The wines consisted of three single varietal wines (Cabernet Sauvignon, Merlot, and Cabernet Franc from the Dr Frank Winery, Finger Lakes, NY, USA) and the three possible 50–50 mixtures of each pair of single varietals. Note that the latter blends must presumably be more chemically complex than the single varietals (see Smith, 2014; Spence & Wang, 2018a, 2018b, Wang & Spence, 2018a, on the notion of complexity in the world of fine wine). Nevertheless, the results revealed that none of the three groups of tasters were able to distinguish the more chemically complex blends from the single varietals at a level that was significantly better than chance (see Campbell, Campbell, & Roberts, 1994; Chadwick & Dudley, 1983; and Smith, Sester, Ballester, & Deroy, 2017, for a similar inability to discriminate single malt whiskies from their blended counterparts).

Given the many well-controlled failures to discriminate wines blind on the basis of their age, quality, or price (see Spence, 2010c, for a review), the question then becomes one of what exactly the wine expert learns when training (see Hughson, 2003; Hughson & Boakes, 2001, 2002, 2009). In a recent review of the literature on perceptual learning, Spence, 2019c; see also Spence & Wang, 2019) there was little published evidence to support changes in sensory threshold accompanying wine expertise (Brand & Brisson, 2012; Parr, Heatherbell, & White, 2002). Rather, the changes that are observed are most apparent in an increased ability to name and categorize wine-relevant aromas (see also Ballester, Patris, Symoneaux, & Valentin, 2008; Brochet & Dubourdieu, 2001; Findlay, Castura, Schlich, & Lesschaeve, 2006; Gawel, 1997; Solomon, 1990, 1991, 1997; Wang, Frank, Houge, Spence, & LaTour, 2019; Wang & Prešern, 2018; Zucco, Carassai, Baroni, & Stevenson, 2011).

Interim summary: studies of perceptual learning in the world of wine suggest that the majority of the learning tends to be more conceptual/cognitive than specifically in terms of changes to sensory thresholds. In part, the reason for this may once again relate to the complexity of the underlying stimulus. Prior studies of perceptual learning in the higher spatial senses of vision, audition, and touch have revealed that the most pronounced perceptual changes are observed under those conditions in which only a single specific attribute of the stimulus is varied across learning trials (Dosher & Lu, 2017). This is hard to achieve in the world of wine given the complexity of this natural product and hence the complexity of the learning environment that it presents (Spence & Wang, 2019).

### Wine marketing

There have been many studies of wine marketing in recent decades. There has been particular interest in both observing and trying to influence the behaviour of shoppers in the wine aisle (e.g., Thach, 2008). In one famous study, North, Hargreaves, and McKendrick (1997, 1999) reported that shoppers in a UK supermarket bought significantly more French (than German) wine when French music was played, whereas they purchased more German wine on those days on which German music was played instead. What is more, of the 44 shoppers who agreed to be questioned after leaving the tills as to why they had chosen to purchase the wine that they had, only 6 shoppers thought that the music playing in the background had influenced their choice. However, while the results of this famous study have been frequently cited over the last 20 years, it is worth bearing in mind that the findings were based on what today can seem like a very small dataset. In fact, a total of only 82 bottles of wine were sold during the 2 weeks in which the study (first published in *Nature*) was conducted (though see Hsu & Chen, in press, for a recent cognitive neuroscience study replicating the priming effect of musical genre on wine selection).

Elsewhere, it has been demonstrated that playing classical music rather than top-40 hits resulted in people spending significantly more money in a North American wine store (Areni & Kim, 1993). A follow-up study that was published the next year by the same pair of researchers (Areni & Kim, 1994) revealed that changing the type of music had a much greater impact on the pattern of wine sales than did changing the level of the ambient lighting. What is true for the sales of wine (in terms of the biasing effect of playing ethnically recognizable or classical music on people’s choice behaviour) has since been replicated in the restaurant/cafeteria context with people’s selection of food (e.g., Yeoh & North, 2010; Zellner, Geller, Lyons, Pyper, & Riaz, 2017; and see Spence, Reinoso-Carvalho, Velasco, & Wang, 2019, for a recent review).

#### The cognitive psychology of wine brands

Many researchers have investigated the impact of the wine label on wine consumer behaviour (e.g., see Boudreaux & Palmer, 2007; Charters, Lockshin, & Unwin, 1999; Cutler, 2006; Gmuer, Siegrist, & Dohle, 2015; Mueller, Lockshin, Saltman, & Blanford, 2010; Shaw, Keeghan, & Hall, 1999; Thomas & Pickering, 2003; Tucker, 1998, for research on wine bottle labels).^3^ Part of the challenge here revolves around the sheer number of different wine brands that are typically available for purchase in any normal wine display/store combined with the fact that many wines change year on year (e.g., Britton, 1992; Rocchi & Stefani, 2005). While a part of the problem for shoppers is in finding the bottle they want, it can also be a challenge to remember/pronounce the name of the wine, even if one remembers what it is that one wants.

Just take the following wines and ask yourself how you would go about trying to pronounce them: Eitelsbacher Karthäuserhofberg Riesling Kabinett*,* Piesporter Goldtröpfchen, and the Hungarian varietal, Cserszegi Fuszeres*.*^4^ As I am sure that you will readily agree, these names lack what the psychologist refers to as “processing fluency” (e.g., Labroo, Dhar, & Schwartz, 2008). Of course, it might well be imagined that a lack of processing fluency in the name of a wine brand would help to set expectations of a more complex tasting experience (see Alter & Oppenheimer, 2006; Dohle & Siegrist, 2014). However, when Gmuer et al. (2015) tested 123 participants in a field study in Switzerland, they found that their participants gave higher hedonic ratings to a wine whose label text was written in an easy as compared to a more difficult-to-read typeface. In this case at least, increased processing fluency (note that the participants had to read the entire wine label before tasting the wine) led to increased liking of the wine. The emerging field of the sound symbolism of luxury brands might also become increasingly relevant here (Pathak, Velasco, Petit, & Calvert, in press).

There has been a great deal of discussion around the rise of the critter brand (e.g., Labroo et al., 2008): think only of all those bottles with a creature, such as an emu, giraffe, cat, frog, etc. prominently displayed on the front wine label. Other wine brands have selected some other distinctive icon, such as a red bicycle, or, in the 1960’s, think of the iconic Blue Nun or Black Tower. Such brands, note, are both easy to remember and easy to pronounce and, so the argument goes, this may have had something to do with their success in the marketplace. Other ways in which the wine marketers have on occasion attempted to make their brand stand out on the shelf, and so capture the shopper’s attention, is by colouring their white wine blue (see Spence, 2018b, for a review) or in one case going so far as to call their white wine “Red”.^5^ There is also evidence that using a downward-pointing triangle on the label (e.g., see the Spanish Izadi wine brand, see http://www.izadi.com/en), may implicitly trigger a danger/fear response, and thus potentially help a brand to stand out from the rest of the bottles on the shelf too (see Velasco, Woods, & Spence, 2015). A separate, but seemingly just as successful approach to wine marketing in recent decades has involved the use of witty wine labels (e.g., Atkin, 2010; May, 2009; Styles, 2004; Williams, 1999), though the consumers’ level of risk aversion may play a role here in determining how successful such an approach is (Lunardo & Rickard, 2019).

It can be argued that the wine aisle represents one of the most challenging of visual search environments. And while it is often claimed that colour drives the consumer’s search for products while shopping in the store (see Spence & Velasco, 2018, for a review),^6^ this would not obviously seem to be the case for wine labels/bottles (with a few notable exceptions; e.g., consider only Campo Viejo’s use of a distinctive yellow label, for a number of their wine brands; see https://www.campoviejo.com/en/wines). The colour and shape of labels influence people’s wine expectations (e.g., Heatherly, Dein, Munafo, & Luckett, 2019; Lick et al., 2017; Lunardo & Livat, 2016). Heatherly et al. recently extended the crossmodal correspondences framework (see Spence, 2011c, 2012) specifically to the design of wine labels. The latter researchers assessed which colours and shapes participants thought were best associated with a Chardonnay wine expressing different aroma characteristics, namely buttery, citrus, floral, smoky, and vegetable. They used projective mapping with 3D shapes (varying in terms of their roundness versus angularity, and also in terms of their complexity - simple versus complex) and colours (red, brown, yellow, and green), along with a wine-label matching study. The results of their projective mapping study revealed that most of the Chardonnay odours were grouped similarly; however, the vegetable-forward Chardonnay wine tended to be associated with sharper shapes. Meanwhile, in Heatherly et al.’s label experiment, yellow labels were better matched with all odours, except the vegetable-forward wine, which was matched to all four colours equally. According to research reported by Lick et al. (2017), red and black wine labels for red wine are most likely to create tangy flavour expectations, while red and orange are most associated with fruity and flowery flavours instead. These flavour expectations based on crossmodal correspondences were stronger in those who bought wine more frequently.

#### The multisensory psychology of wine bottles

Piqueras-Fiszman and Spence (2012) conducted a field study demonstrating correlation between the weight of the wine bottle and the price across all of the 275 bottles for sale in a branch of the Oxford Wine Company store in Oxford.^7^ They found that the consumer pays an average of £1 more for each 8 g extra weight of glass, or should that be that they obtain an average of 8 g more weight of glass for every £1 extra they pay? There is undoubtedly an implicit suggestion amongst the wine press that wine makers use this product-extrinsic cue deliberately (e.g., see Goldstein & Herschkowitsch, 2010).^8^ It is the presence of additional weight that may also help to explain why so many people prefer drinks from a bottle rather than from a can (see Barnett, Velasco & Spence, 2016; Lefebvre & Orlowski, 2019; see also Kampfer, Leischnig, Ivens, & Spence, 2017). While Old World wine producers are often restricted to certain iconic bottle designs (and hence the shape/weight is more or less fixed), New World wine producers have rather more freedom to play with their bottle designs. In fact, certain wine bottles weigh more than 2 kg when empty, whereas other cheaper wines weigh less than 1 kg when full (see also Spence, 2017a). The depth of the punt is also an intriguing feature here, though not one that has received any empirical research as far as I am aware. That said, this feature, and its possible association with wine quality is one that has attracted more than its fair share of discussion online (e.g., Anon, 2015; Touzalin, 2015).

Spence and Wang (2017) recently demonstrated that the sound of closure - cork versus screw-cap - can also help to set specific product-related expectations in the mind of the wine consumer (cf. Piqueras-Fiszman & Spence, 2015; Wang & Spence, 2019). They had 140 individuals (with a range of levels of wine expertise) taste and rate four glasses of red wine blind. Unbeknownst to the participants, they actually sampled two reasonably similar Argentinian wines (a Terrazas de los Andes, Malbec 2015, and a Catena, Malbec 2015) twice. On one occasion, one of the wines was tasted after hearing the sound of a cork-stoppered wine bottle being opened while the other was sampled after hearing the sound of the crack of the opening of a screw-top bottle. The second time that the wines were evaluated, the participants had to uncork one wine bottle and open the screw-top of another bottle, so the sound that they heard was self-generated. However, regardless of whether the participants only heard the sound, or else performed the action that gave rise to the packaging opening sound that they heard, they rated the wine as being of higher quality when it appeared to have come from a cork-stoppered bottle. The participants also rated the wine as being more appropriate for a celebratory occasion, and they rated their own mood as more celebratory, after hearing the sound of the wine bottle’s cork pop. Ratings of the intensity of the wine were, however, unaffected by the experimental manipulation.^9^ Such results should perhaps not come as such a surprise given that knowledge of closure type, no matter the sense by which that information is transferred has been documented to influence consumers (e.g., Marin et al., 2007; Marin & Durham, 2007; Reynolds, Rahman, Bernard, & Holbrook, 2018).

Interim summary: the wine aisle is more complex and dynamically changing than perhaps any other category of branded food or beverage product. In part this reflects the natural variation of this product that changes year on year, together with the multitude of small brands/producers. In fact, according to wine writer Natalie MacLean (2008): “There are more than a million producers, and each one makes at least a few wines, all of which change every year. Multiply that together and it’s dazzling, overwhelming and confusing.” (quoted in Black, 2008). While on the one hand, this obviously makes the wine-taster’s job much harder on blind tasting, at least as far as identifying specific wines, when it comes to the regular consumer purchasing wine in the store, the marketers have had to be more inventive in terms of standing out from the constantly changing competition on the wine shelf (see also Chaney, 2000). This has led to a variety of innovative marketing strategies that can potentially be applied to other, less-developed, product categories. At the same time, however, the wine bottle itself, along with its traditional cork stopper, represents a very powerful marketing tool, especially for those who are able to caricature specific design features, such as, for example, increasing the weight of the bottle to try and signal quality (Spence, 2019e; Velasco & Spence, 2019). Selling the wine in a box can also help connote quality (Sung, Crawford, Teah, Stankovic, & Phau, 2020).

### The wine glass

Whenever we drink, there is always a receptacle, be it a glass, beaker, mug, bottle, or can. And while there has been a great deal of research on the sensory/chemical (what some have termed the organoleptic^10^) properties of the drink itself, there has been far less research on the impact of the receptacle on the tasting experience (see Spence & Wan, 2016, for a review). Until very recently, the only exception to this generalization came from the world of wine. In fact, over the last half century or so at least 20 studies have been published assessing the impact of the shape/size of the wine glass on people’s rating of the aroma/bouquet and taste/flavour of wine (see Spence, 2011b, for a review).^11^ The research clearly shows that if the taster does not know which wine glass they are evaluating a wine in/from, either because they have been blindfolded while the glassware is agitated under the taster’s nose (Cliff, 2001; Delwiche & Pelchat, 2002) or because the glass in which the taster evaluates the wine is different from the glass in which the wine has been allowed to breathe (Russell, Zivanovic, Morris, Penfield, & Weiss, 2005), the glassware seems to make little difference to the taster’s experience. However, as soon as the latter become aware of the nature of the glass from which they are tasting, the glassware can suddenly make a huge difference to the tasting experience (e.g., Fischer, 1996; Fischer & Loewe-Stanienda, 1999; Hummel et al., 2003; Venturi et al., 2014; Venturi et al., 2016; Vilanova, Vidal, & Cortes, 2008; see also Attwood, Scott-Samuel, Stothart, & Munafò, 2012; Manska, 2018).

What such results suggest is that the influence of the wine glass is more psychological than physico-chemical in origin. Hence, the available research argues against the suggestion that the specific shape of the glass changes the flow properties of the liquid across the tongue (or rather, if it does, it argues against this having a noticeable difference to the tasting experience), or that the shape of the headspace above the wine in the glass helps to concentrate specific aroma volatiles. Instead, one might more fruitfully want to consider the crossmodal correspondences that are invoked by the shape of the glass itself (e.g., see Spence & Deroy, 2012, Spence & Deroy, 2013b, 2013c, 2013d; Velasco, Woods, Marks, Cheok, & Spence, 2016), and the more semantic associations with the apparent quality of the glassware (Billing, Öström, & Lagerbielke, 2008). Here it is interesting to note that while a round (rather than straight-sided) glass has been shown to bring out the fruity and sweet notes in both wine (Hummel et al., 2003) and beer (Mirabito, Oliphant, Van Doorn, Watson, & Spence, 2017), simply coating the outside of a 3D-printed cup with round versus angular macrotextural features has a similar effect on the perceived sweetness of a drink (Van Rompay, Finger, Saakes, & Fenko, 2017; though see also Machiels, 2018). Notice how, in the latter case, the flow properties of the liquid in the glasses will have been indistinguishable.

While sensitive measurement devices can often detect differences in the concentration of volatile aromas in the headspace above the surface of the wine in the glass (e.g., Arakawa et al., 2015; Hirson, Heymann, & Ebeler, 2012; Liger-Belair, Bourget, Pron, Polidori, & Cilindre, 2012), that does not necessarily mean that the taster can pick up on such differences. One of the important factors to bear in mind here is also the fact that mechanisms of olfactory perceptual constancy may well work against any attempts to maximize the volatile odorant intensity (Spence, 2017a; see also Russell et al., 2005). That is, just like for the other senses, it has been suggested that mechanisms of perceptual constancy are involved in the olfactory system to try and obtain a better estimate of the odour source without being unduly swayed by other factors, such as any change in nasal airflow (see Spence, 2017a; Teghtsoonian, Teghtsoonian, Berglund, & Berglund, 1978).

Interim summary: the drinking vessel, be it glass, goblet, cup, mug, beaker, or whatever else is always there when we drink (see Spence, 2011b, for a review), just as the flatware is an ever-present aspect of our dining experiences (Spence, 2017a). However, while glassware it is an important factor influencing the multisensory drinking experience it has to date been little studied outside the world of wine. A careful assessment of the literature on the wine glass reveals that it can exert a profound influence over the tasting experience but that the origins of such effects are more psychological than physico-chemical in origin. Recently, researchers have started to assess the psychological influence of the drinking vessel in other beverage categories too. That said, the perceptually complex, and temporally evolving nature of the experience of fine wine may mean that the influence of glassware may ultimately be easier to observe there than in the case of a simpler beverage such as cola, or water (see Spence & Wan, 2016, for a review).

### Multisensory atmospherics and the wine-tasting experience

Spence et al. (2014) conducted what is perhaps the largest experimental wine-tasting event ever conducted, building on earlier research by Oberfeld, Hecht, Allendorf, and Wickelmaier (2009); see also Ross, Bohlscheid, & Weller, 2008; Sauvageot & Struillou, 1997. Spence and his colleagues had almost 3000 members of the general public taste and rate a single 100-ml glass of Campo Viejo Reserva 2008 Rioja red wine under four different ambient conditions, involving regular white lighting followed by either red or green lighting. Both of the latter two coloured-lighting conditions were then presented together with putatively “sweet” or “sour” music, respectively (based on prior sonic seasoning research by Knöferle, Woods, Käppler, & Spence, 2015). The participants were given a scorecard on which they rated on line scales “How fruity vs. fresh does the wine taste?”, “How intense the flavour?” and “How much do you like the wine?”

The results revealed that the wine was rated as tasting significantly fruitier under red than under green lighting, and with putatively sweet rather than with sour background music. Interestingly, the scorecard on which the participants entered their responses also had a small space for those who took part to reflect on their experience should they so desire: 85 of the participants chose to write something in this space. The first-person reports helped to highlight the striking nature of the change in tasting experience that some of the participants experienced on sampling the wine under the different environmental conditions: “Fantastic experience. Really interesting. Changed perceptions completely.”; “Yes, amazing experience.”; “Great experience. I didn't think the colour/sound would alter my perception as much as that!” (quotes from Spence et al., 2014, pp. 9–10).

That said, not everyone has demonstrated an impact of the environment on the wine-tasting experience. For instance, Jiang, Niimi, Ristic, and Bastian (2017) reported that changing the visual atmospherics in a room (introducing flowers, pictures, and coloured lighting) had no effect on 105 wine consumers’ ratings of a red wine. In the latter study, one atmospheric condition had a floral theme, the other, a “green” theme, with the researchers wanting to know whether they could bring out the floral or green notes in the three Cabernet Sauvignon wines that the participants were to taste. It is, at present, unclear why the results of this study should have been different from others in this space. Nevertheless, taken together, the admittedly limited evidence published to date clearly supports the view that the multisensory atmosphere can influence people’s taste/flavour perception, their choice behaviour, and even how much they end up consuming, be it in the world of wine, or when considering other alcoholic beverages (e.g., Sester et al., 2013; Velasco, Jones, King, & Spence, 2013; Wang & Spence, 2015b).

Interim summary: while Spence et al. (2014) favoured a direct crossmodal perceptual account of the influence of lighting colour on their participants’ taste/flavour perceptions it should be noted that an emotional mediation account has also been suggested by other researchers working in the area. For instance, Oberfeld et al. (2009), p. 807 had the following to say when attempting to explain their earlier results of changing the colour of the lighting in a German winery: “if a colour induces a positive mood or emotion […] then the same wine tasted in this positive mood is liked better than when in a negative mood”*.* Once again, the contextual effects on the wine-tasting experience are likely to be replicated in the case of other food and drink products and in some cases already have (see Wang & Spence, 2015b).

At this point in proceedings it is perhaps also worth pausing to highlight the fact that the consumption of significant amounts of alcohol has been reported to give rise to a change in perception of those of the opposite sex. This effect is colloquially known as the “beer goggles” effect (e.g., Gladue & Delaney, 1990; Jones, Jones, Thomas, & Piper, 2003). The effect appears to be strongest when men under the influence of alcohol judge the attractiveness of less attractive to moderately attractive women. However, I am not aware of a similar effect on the perception of wine as has been proposed previously in the case of attractiveness judgements. That being said, moderate amounts of alcohol have been shown to result in people rating landscape paintings as more attractive under the influence of moderate amounts of alcohol (Chen, Wang, Yang, & Chen, 2014; and see Fretter, 1971, on the notion of wine as art). What is also worth stressing at this point is that the amount of alcohol consumed in many of these experiential wine-tasting events tends to be minimal - e.g., in The Campo Viejo Colour lab each participant only received one small measure of wine throughout the entire experience. This contrasts the beer goggles effect, which is typically observed under conditions of heavy consumption (see also George, Rogers, & Duka, 2005, for the acute effects of alcohol on decision making).

### Musical crossmodal correspondences with wine

Traditionally, the suggestion was that professional wine tastings take place in silence (e.g., Peynaud, 1987). However, the last decade or two has seen a veritable growth of interest in the matching of music and wine, and the crossmodal influence of the former on the latter (see Spence & Wang, 2015a, 2015b, 2015c, for reviews). Such sonic seasoning or oenosthesia research has run in parallel to an emerging body of laboratory research documenting first that people intuitively match basic tastes, such as sweet, sour, bitter, and salty with particular musical attributes, and thereafter that playing music (or soundscapes) with matching or mismatching sonic properties can influence people’s ratings of the taste/flavour of a variety of foods (e.g., Bronner, Bruhn, Hirt, & Piper, 2012; Crisinel & Spence, 2010, 2012; Hauck & Hecht, 2019; Höchenberger & Ohla, 2019; Knöferle et al., 2015; Kontukoski et al., 2015; Mesz, Sigman, & Trevisan, 2012; Mesz, Trevisan, & Sigman, 2011; Simner, Cuskley, & Kirby, 2010).

There is, by now, an extensive body of research on crossmodal correspondences between wine and music (see Spence & Wang, 2015a, 2015b, 2015c, for reviews). This makes sense inasmuch as the experience of both music and wine are temporally evolving and often described as “complex”. What is more, the terms used to describe olfactory stimuli and musical notes sometimes overlap. Consider for instance here only the use of the terms such as “high notes”, “low notes”, “chords”, and “harmony”, etc. (see Deroy, Crisinel, & Spence, 2013, for a review). Numerous studies have demonstrated that people consistently match certain wines with specific pieces of music, at least under those conditions in which they are forced to make a choice (Spence et al., 2013; Wang & Spence, 2015a; see Spence & Wang, 2015a, for a review).

In one study, Spence et al. (2013) demonstrated that Domaine Didier Dagueneau Pouilly Fumé, a crisp white wine, was rated by participants as matching Mozart’s Flute Quartet in D major - Movement 1 significantly better than Tchaikovsky’s String Quartet No 1 - Movement 2. However, the reverse pattern of results - that is, a better match with Tchaikovsky than Mozart - was observed when participants tasted a glass of Chateau Margaux, a rich red Bordeaux wine instead. Furthermore, in a subsequent experiment, tasting the wines while listening to matching music resulted in a small but significant increase in people’s rated enjoyment of the wine-drinking experience as compared to tasting the same wines in silence (Spence et al., 2013).

Summarizing the literature on wine-music correspondences, it is worth highlighting the fact that both simple and complex crossmodal correspondences have been demonstrated (see Spence, 2011c, 2018a, 2019d; Spence et al., 2013). Indeed, the importance of emotion to such crossmodal matching is hinted at by the following quote from Paul White: “Red wines need either minor key or they need music that has negative emotion. They don’t like happy music…Cabernets like angry music.” (Gray, 2007a). The emotional mediation of crossmodal correspondences between sound and basic taste has also been reported by Wang, Wang, & Spence, 2016; see also Crawshaw, A. (2012). How musical emotion may provide clues for understanding the observed impact of music on gustatory and olfactory perception in the context of wine-tasting. Unpublished manuscript; Wang & Spence, 2017a). The emotional mediation account of music’s influence on wine-tasting is in some sense analogous to the way in which Oberfeld et al. (2009) attempted to account for the effect of strongly coloured lighting on the wine-tasting experience, as mentioned earlier.

One of the reasons as to why wine might represent an especially good subject for empirical research that capitalizes on the crossmodal correspondences is that it is often both complex, in terms of multiple elements in a temporally evolving tasting experience, and also is a product that displays infinite variation. What this means in practice is that it is going to be harder for a participant or consumer to fix on a specific flavour memory as, for example, might be possible were one to use a branded product such as Coca-Cola, say. The research shows that wine experts are also affected by music. Some of the earliest reports on the modulatory effects of music on wine-tasting actually have come from wine makers (see Gray, 2007a, 2007b).

In a study conducted at the International Cool Climate Wine conference (Wang & Spence, 2017b), 154 very experienced wine tasters - the majority of whom were professionals working in the wine business (with an average of 18.1 years of experience) - were tested. The first study assessed the impact of putatively “sweet” and “sour” soundtracks on taste evaluation,^12^ whereas the second study assessed more subtle wine-specific terminology such as length, balance, and body. The results revealed that a crossmodal influence of music on wine perception can indeed be demonstrated in wine experts.

When thinking about music’s influence on the tasting experience, it can be helpful to discriminate between four different kinds of judgements, or impressions that we may ascribe to a wine (see Spence & Wang, 2015c): hedonic - how much do we like the wine? Sensory - our assessment of the physical properties of the wine (such as its sweetness, acidity, alcohol) and their impact on the drinker (astringency, length, etc.); analytic - concerning such attributes as age, complexity, balance, quality, and price assessment; and descriptive - would one describe the wine as heavy or light, zingy or lush, masculine or feminine? Music can potentially influence all four kinds of judgements. It is, however, an open question as to whether all four kinds of judgement are equally susceptible to the influence of musical interventions.

One issue that has yet to be resolved in the world of sonic seasoning is whether wine experts are influenced as much by music as are social drinkers. Relevant here, Wang and Spence (2017b) found no relationship between wine expertise, as measured in years of wine-tasting experience, and the magnitude of the influence of music on their participants’ wine ratings. Another issue that has yet to be fully resolved concerns whether the influence of music (and/or other soundscapes) are more pronounced under those conditions in which the taster’s attention has somehow been drawn to the potential correspondence between what they are listening to and what they are tasting, or whether such effects can occur in the absence of a specific connection being made (see Spence, 2019a).

To date, those researchers interested in wine-music pairing have typically picked pre-existing musical selections, very often instrumental classical music in order to accentuate a specific attribute in the wine (e.g., see Spence, 2011a; Spence et al., 2014; Wang & Spence, 2015a, 2015b; White, 2008, though see De Luca, Campo, & Lee, 2019; Gray, 2007a, 2007b; North, 2012, for exceptions). For example, North (2012) conducted a study showing that background music can be used to prime, and hence bias, attributes of the tasting experience, such as assessments of how “powerful and heavy” or “zingy and refreshing” a wine appears to be. North had 250 students studying in Scotland evaluate a glass of either white or red wine, while at the same time listening to music that had been pre-determined to be associated with one of four metaphorical categories (“powerful and heavy”, “zingy and refreshing”, “subtle and refined”, or “mellow and soft”). The students’ judgements of the wine were influenced by the music, with the students rating both wines as tasting more powerful and heavy when listening to Carmina Burana by Karl Orff and as tasting more zingy and refreshing when listening to Just Can’t get Enough by Nouvelle Vague (though see Spence & Deroy, 2013b, on the most appropriate interpretation of these results).

Burzynska, Wang, Spence, and Bastian (2019) recently published the results of research in which they attempted to enhance the perception of the mouthfeel character (of body and palate weight) in a wine simply by having people taste wine while listening to a low-frequency note (10–200 Hz). In particular, 50 participants (including 19 wine novices and 31 individuals with some experience of wine tasting) took part in the study in which all of the participants tasted two similar wines, a New Zealand Pinot Noir and a Spanish Garnacha. The wines were tasted in silence, together with a 100-Hz (bass) note (approximately equivalent to a musical note G_2_), and while listening to a relatively higher pitch 1000-Hz sine wave tone (approximately equivalent to a musical note B_5_). The participants had to rate the body of the wine and evaluate its aromatic intensity and acidity, and their liking of it. Listening to the bass note resulted in the Pinot Noir wine being rated as significantly fuller-bodied when tasted with a bass frequency than in silence or with a higher-frequency sound. Listening to the bass note also resulted in the other wine, the Spanish Garnacha, being rated as significantly more aromatically intense than when tasted in the presence of the higher-frequency auditory stimulus.

Wang, Frank, Houge, Spence, and LaTour (2019) recently introduced music as a unique aspect of a VIP tasting room experience at a family-owned Finger Lakes winery. A convenience sample of 46 participants (considered as “wine enthusiasts”) tasted four oaked still wines (two white and two red from the host vineyard; namely 2015 Chardonnay, the 2014 Hilda Chardonnay, the 2014 Pinot Noir, and the 2014 Cabernet Sauvignon) in silence and with a complementary soundtrack (the soundtrack can be streamed at https://soundcloud.com/benhouge/chivas-bitter),^13^ and rated the fruitiness, spiciness, and smoothness of each wine in both sound conditions. The results revealed that the wines tasted while the soundtracks were playing in the background were rated as significantly fruitier and smoother than the same wines when tasted in silence. There was, however, no effect on spiciness ratings.

#### Extraordinary wine-tasting experiences

Finally here, it is interesting to note that there is growing interest not just in modifying tasters’ ratings of wine attributes such as fruitiness, acidity, or sweetness, but in actually delivering extraordinary tasting experiences that are somehow more (or greater) than the sum of their parts (Spence, 2020a; see also Mitchell et al., 2017). There have, for instance, been occasional reports of people being brought to tears by the combination of wine and purposely composed matching music (e.g., Knapton, 2015). Elsewhere, one finds descriptions such as the following from James John, Director of the Bath Wine School, talking about tasting Chardonnay while listening to Mozart’s Laudate Dominum: “[…] Just as the sonant complexity is doubled, the gustatory effects of ripe fruit on toasted vanilla explode on the palate and the appreciation of both is taken to an entirely new level” (quoted in Sachse-Weinert, 2012). The possibility of delivering such extraordinary multisensory tasting experiences by carefully combining music and wine tasting providing one answer to the refrain that is sometimes heard (especially, it would seem, from Masters of Wine) concerning why one should bother with changing the taste of wine via musical accompaniment when one could just pick a different wine in the first place (e.g., Spence, 2020a; Spence & Wang, 2015c).

At the same time, however, Sachse-Weinert’s (2012) quote might also make one wonder how much of what goes on in, and is written about, the world of wine is some kind of social construction based on expectations rather than necessarily reflecting a genuine perceptual effect. The results of blind tastings (see Spence, 2010c, for a review), together with the extensive literature on the absence of sensory threshold changes in expert wine-tasters (see Spence, 2019c; Spence & Wang, 2019) certainly do suggest that higher-level cognitive/conceptual constructs, together with the associated mental imagery concerning what one is expected to taste/experience play a major part in the wine-tasting experience, especially for the experts (and here, I am thinking particularly about the wine writers; see also Bach, 2007).

Tasting a quality wine will likely reveal a temporally evolving range of flavours and oral-somatosensory attributes to the palate of the attentive wine taster (as revealed using sensory analysis techniques such as the temporal dominance of sensations (TDS, e.g., Meillon, Urbano, & Schlich, 2009; Wang, Mesz, et al., 2019). As such, selecting music to match just one attribute of the wine-tasting experience can sometimes be less than ideal. It should come as little surprise, therefore, to find that researchers have recently started to compose music that evolves in synchrony with the specific attributes that tasters are likely to detect in a wine (see Crisinel, Jacquier, Deroy, & Spence, 2013, for a similar earlier approach to cognac, and https://www.youtube.com/watch?v=rph6oyIEJ9o, for a recent project conducted together with Godiva chocolate).

However, while all of the sonic seasoning research in the world of wine is impressive in terms of the changes in people’s ratings of specific wine attributes, it is worth remembering that the largest improvement to people’s enjoyment of the wine they are drinking may come about from pairing with music that they enjoy listening to (see Reinoso-Carvalho, Dakduk, Wagemans, & Spence, 2019, for evidence consistent with this claim from beer-music pairing studies; see also Cramb, 2008).

Interim summary: in recent years, there has been a rapid growth of interest in the multiple ways in which what we hear may influence our perception of wine, and the wine-drinking experience more generally (see Spence & Wang, 2015a, 2015b, 2015c, for reviews). While the emotion that may be associated with, or induced by, listening to music may explain some of the effects that have been documented, a more direct perceptual effect resulting from crossmodally corresponding sounds drawing the taster’s attention to something in their tasting experience that they might otherwise have neglected would also seem to be an important part of sonic seasoning (Hennion, 2015; see Spence, 2019a; Spence et al., 2019, for reviews). It has been suggested that the complex and temporally evolving nature of the experience that is typically associated with drinking a quality wine might make such attentional effects more obviously than in the case of a simpler taste experience. Recently, researchers have started to compose music specifically to match the likely temporal evolution of the tasting experience. In order to do this, sensory science techniques, such as TDS, that allow one to track the ebb and flow of different notes, or elements, in the tasting experience over time have proved to be very helpful (Wang, Mesz, et al., 2019).

## Conclusions

Given the emerging understanding of wine psychology, broadly defined, it should come as little surprise to see the explosion of experimental multisensory wine marketing research that has been published in recent years (see Spence, 2019b, for a review).^14^ Wine makers and wine brands, both large and small, are increasingly coming to recognize the benefits of cognitive research to help enhance their cellar door and tasting room experiences for discerning customers (see Spence et al., 2014; Wang, Frank, et al., 2019). As should have become apparent, crossmodal correspondences between wine and shapes, colours (Heatherly et al., 2019), musical stimuli (e.g., Burzynska et al., 2019; Wang, Mesz, et al., 2019), and even tactile stimuli (see Wang & Spence, 2018b)^15^ are potentially relevant when trying to communicate with the consumer, be it in the store or while tasting in the home or elsewhere.

There is, in other words, a potentially rich interaction between business-relevant findings that can also generate insights that should be of interest to basic researchers. Understanding the many influences on the wine-tasting experience may provide relevant insights for various areas of cognitive research. Importantly, however, there are also grounds for believing that our understanding of basic issues in cognitive research can be furthered by studying a perceptually, not to mention chemically complex, culturally and historically ecologically valid foodstuff such as wine (e.g., Estreicher, 2006; Unwin, 1996). Returning to a point that was made in the introduction, one can see this approach in terms of Rozin’s (2006) notion of domain-led rather than process-driven approach to areas of interest to those working in applied psychology. And, for those interested in the world of food and drink more generally, there is a sense in which it makes sense to ask what has been done/discussed/discovered in the world of wine before progressing one’s specific research agenda. Indeed, the latest research on everything from the impact of the colour and shape of labels/packaging (de Sousa, Carvalho, & Pereira, in press; Pelet, Durrieu, Lick, 2020) through to the impact of felt texture on the tasting experience in the world of coffee (Carvalho, Moksunova, & Spence, 2020; Pramudya, Choudhury, Zou, & Seo, 2020) would appear to support the previous findings that have already been established in the world of wine (e.g., Heatherly et al., 2019; Wang & Spence, 2018b).

One of the issues that undoubtedly adds to the complexity of wine choice is that it is often consumed together with food, and there is a growing literature on the art and science of flavour pairing as in the case of food and wine matching (e.g., Hall, Lockshin, & O’Mahony, 2001; see Spence, 2020b, for a review). In fact, one of the factors that can make matching food and wine so challenging is the existence of perceptual interactions between the tastes that are present in the component foods, such as mixture suppression, adaptation, and release from masking (e.g., Breslin & Beauchamp, 1997; Dubow & Childs, 1998; McBurney & Pfaffmann, 1963; and see Spence, n.d., submitted; Wang, Mesz, et al., 2019, for reviews). It is currently an open question as to whether any of these intramodal interaction effects can be fruitfully extended to the crossmodal case, such as to provide an explanation for sonic seasoning. At the same time, however, it should be remembered that certain of these phenomena, such as masking, may be restricted in the intramodal case (Gescheider & Niblette, 1967; see also Hillis, Ernst, Banks, & Landy, 2002).

While wine research undoubtedly has a number of practical applications, it also provides insights concerning multisensory perception that are relevant to basic scientists. For instance, laboratory researchers interested in various aspects of multisensory integration, such as the audiovisual ventriloquism effect or visuotactile interactions in shape or orientation perception, typically present the various modalities simultaneously (e.g., Alais & Burr, 2004; Ernst & Banks, 2002; Gori, Del Viva, Sandini, & Burr, 2008). However, what becomes clear from the case of wine-tasting is the sequential nature of the taster’s multisensory interaction with the product. First come the visual cues, then orthonasal olfaction, and thereafter gustation, oral-somatosensation, and eventually retronasal olfaction. As such, the earlier presented cues (e.g., vision) tend to set expectations, and perhaps even generate crossmodal mental imagery concerning the tastes and flavours that are expected to be present in the wine (Nanay, 2018; Piqueras-Fiszman & Spence, 2015; Spence & Deroy, 2013d). These sensory expectations then anchor, guide, and possibly interact with the subsequent sensory inputs in ways that are yet to be fully elucidated.

## Data Availability

Not applicable.

## Footnotes

1 The colour of the wine still influences participants’ judgements even when the latter are instructed to ignore the visual cues because they may be misleading (see Parr et al., 2003; see also Zampini, Sanabria, Phillips, & Spence, 2007, Zampini, Wantling, Phillips, & Spence, 2008, for similar results with fruit-flavoured soft drinks).

2 One of the challenges associated with working with wine is the suggestion that bottle variation can lead to significant differences between bottles of the same wine. What is more, the fact that the product is in a slow yet constant state of evolution (e.g., Lee, Kang, & Park, 2011; Wirth et al., et al., 2012), means that it is next to impossible to replicate exactly the conditions/stimuli in any previous published study.

3 As wine label designer, Dave Osmundson (quoted in Cutler, 2006) notes: “With some of these bigger companies that are selling lower end, value wines, if you were to taste the wines, you would see little difference, but the label plays even a bigger role with the package. When you are making millions of cases of wine, it's hard to make that wine taste unique. The package makes the difference: the identification with those colors, that design.”

4 According to May (2009), the correct pronunciation for the latter crossing of Gewurtztraminer and Irsai Oliver grapes from the Neszmely winery in Hungary is “Chair-Sheggy Foo-share-us”.

5 This presumably triggering some kind of Stroop-like interference (Stroop, 1935; see also Velasco, Wan, et al., 2015).

6 For instance, Singh (2006, p. 783) claims that colour drives 62–90% of all consumer purchasing decisions (see also Swientek, 2001).

7 Wine, like lipstick, is one of the few products where the volume/mass of the product itself is essentially fixed (750 ml for the majority of wine bottles), hence meaning that any changes in packaging weight are perhaps more noticeable than they might otherwise be for other product categories.

8 For instance, Goldstein and Herschkowitsch (2010, p. 80) claim that: “These Bogle bottles are hefty, and their weight is a nice feature – one that often tricks people into thinking the wine is more expensive than it really is.”

9 This study was conducted in the UK. It is possible that the results may have been different if the study were to be repeated in New World wine growing regions, such as Australia or California, where quality wines are often sold in screw-top bottles (Marin, Jorgensen, Kennedy, & Ferrier, 2007; Taber, 2007).

10 Organoleptic properties are defined as those aspects of food and drink that create an individual experience via the senses - including taste, sight, smell, and touch (see Bruni, 2011).

11 According to Hummel et al. (2003, p. 197): “Many wine connoisseurs claim that the shape of the glass exerts a direct impact on the taste of wines. In fact, many agree that the glass itself may change intensity, quality, and hedonic tone of a given wine.”

12 The sweet soundtrack had bells, piano, and synthesizer, has consonant harmonies, and legato articulation. The sour soundtrack, by contrast, included piccolo and clarinet, has dissonant harmony, and staccato articulation. The soundtracks from both studies can be heard at https://soundcloud.com/janicewang09/sets/iccws-2016.

13 The instruments were cello, woodblocks, temple bells, some low percussion, a sustained woodwind texture, and a low drone sound comprising several elements (including voice and string instruments; Houge, 2015).

14 In fact, there is even an app now that allows one to scan a wine label and it will select what is promised to be matching music (http://winelistening.com/; see also Jones, 2014; https://sabordasmusicas.withspotify.com/).

15 The Italian Futurists had mentioned something very similar, which they referred to as *syn-tactilismo* (Marinetti, 1932/2014).
